# Supplementary material for: Perturbation of Autophagy by a Beclin 1-Targeting Stapled Peptide Induces Mitochondria Stress and Inhibits Proliferation of Pancreatic Cancer Cells
Source: Cancers (Basel). 2023 Feb 2;15(3):953. doi: 10.3390/cancers15030953 (PMC9913477; doi:10.3390/cancers15030953)

Figure 1B (repeat 3 times)

Trial 1

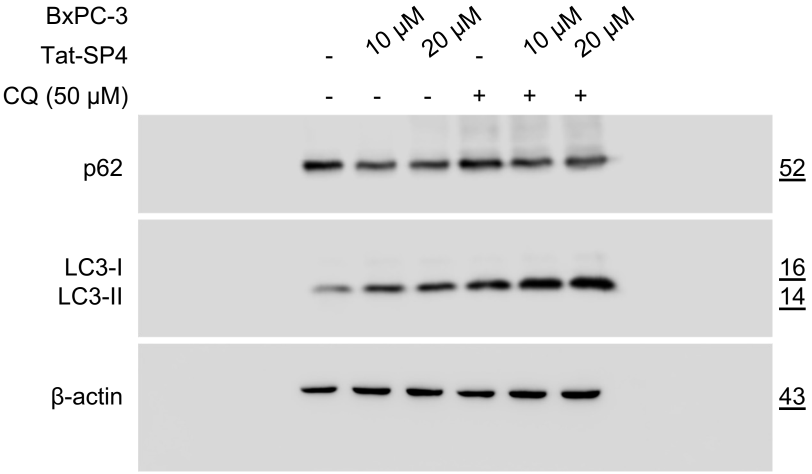

Trial 2

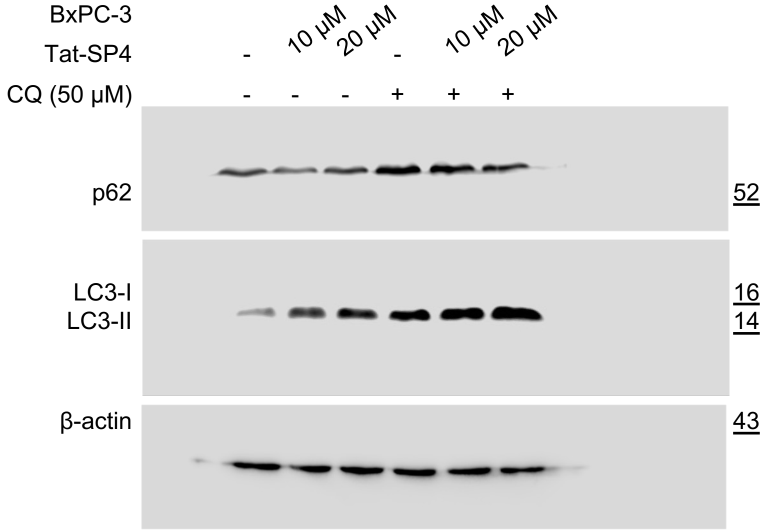

Trial 3

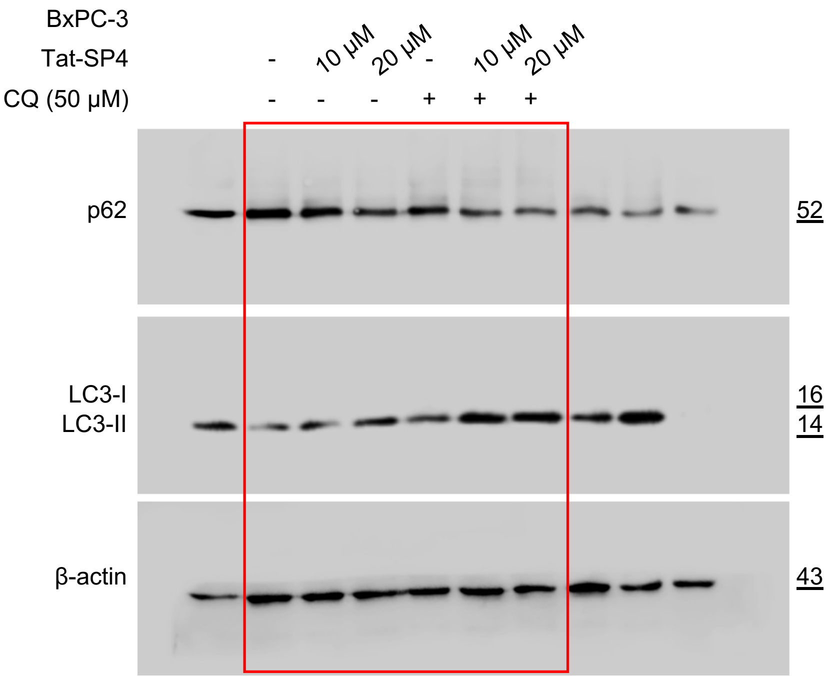

Figure 1D (repeat 3 times)

Trial 1

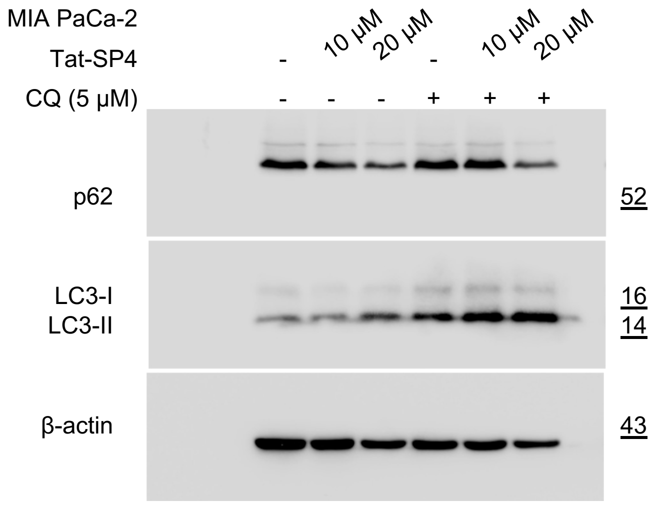

Trial 2

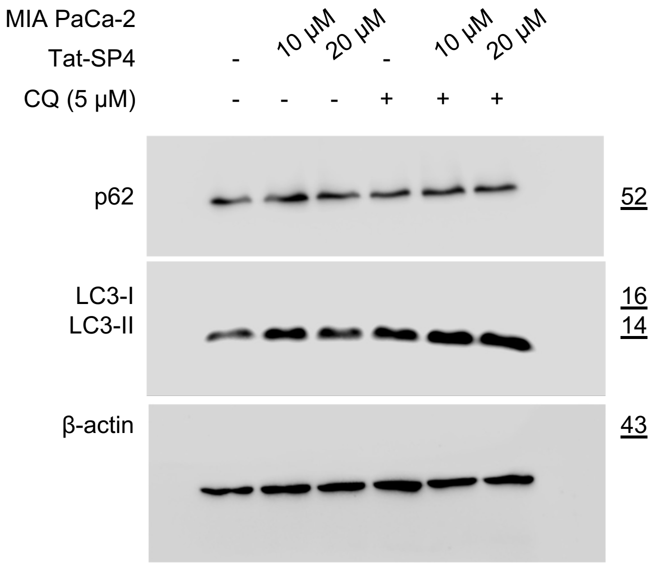

Trial 3

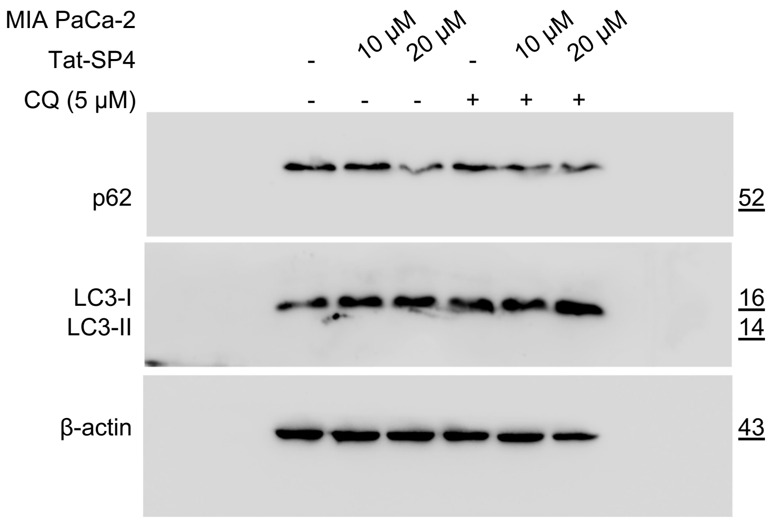

Figure 1F (repeat 3 times)

Trial 1

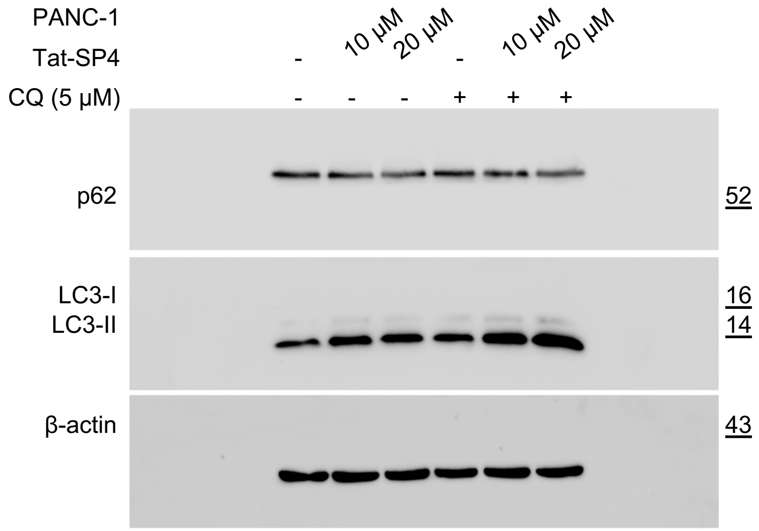

Trial 2

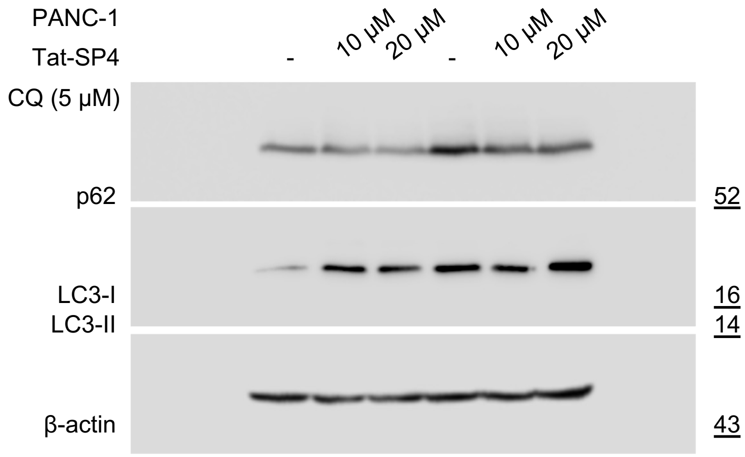

Trial 3

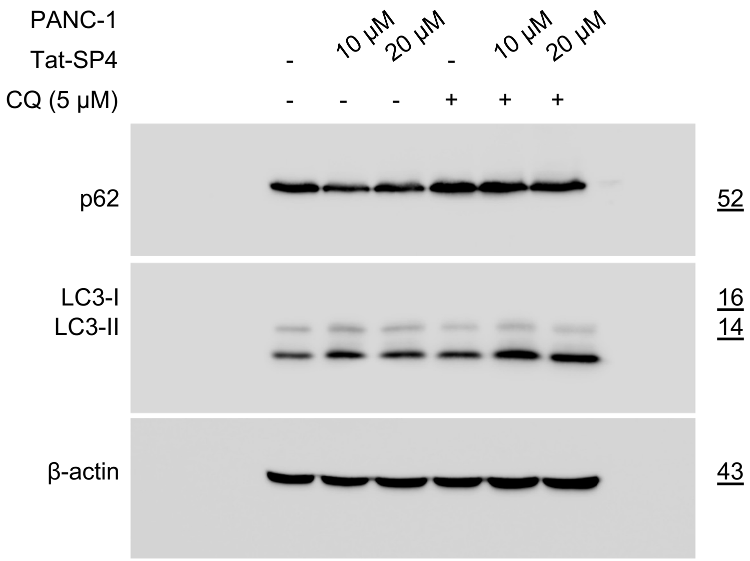

Figure 2A (repeat 3 times)

Trial 1

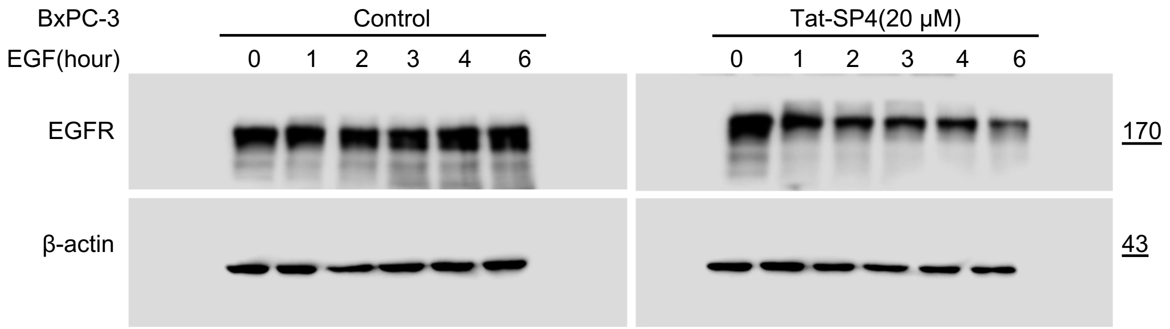

Trial 2

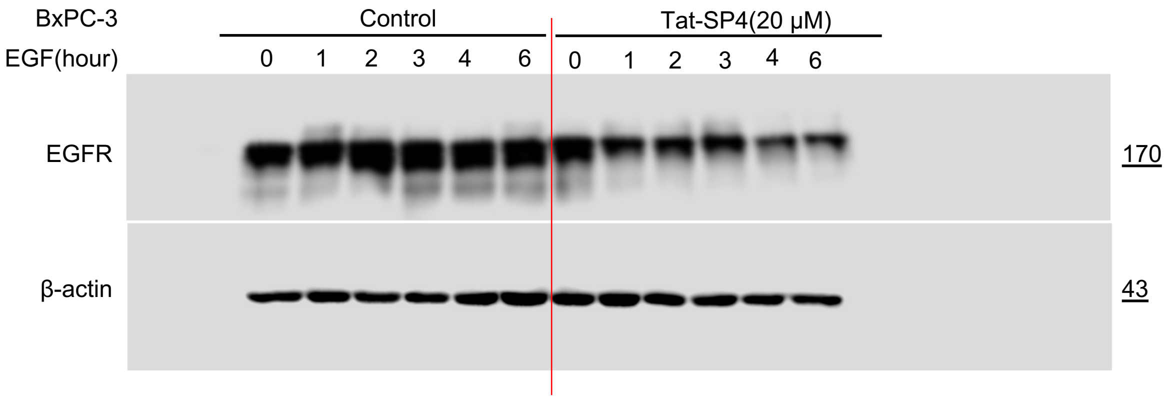

Trial 3

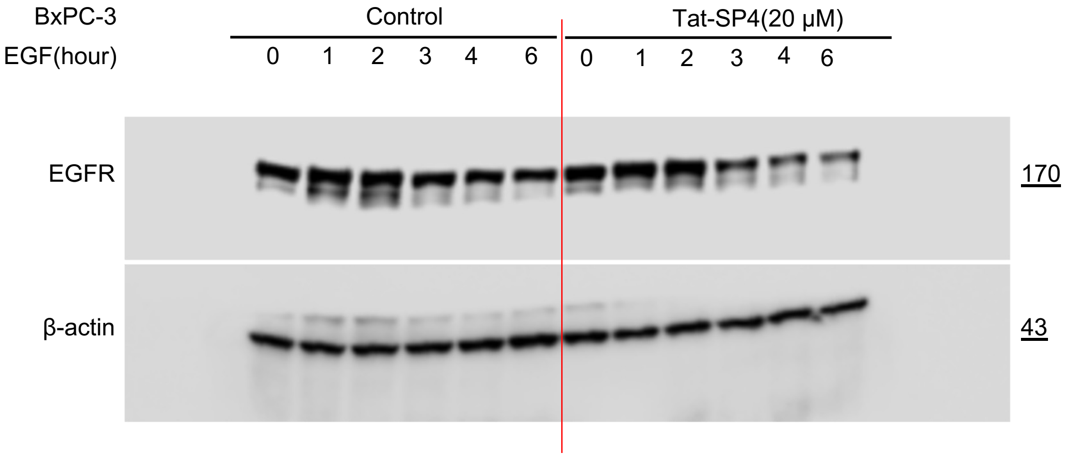

Figure 2C (repeat 3 times)

Trial 1

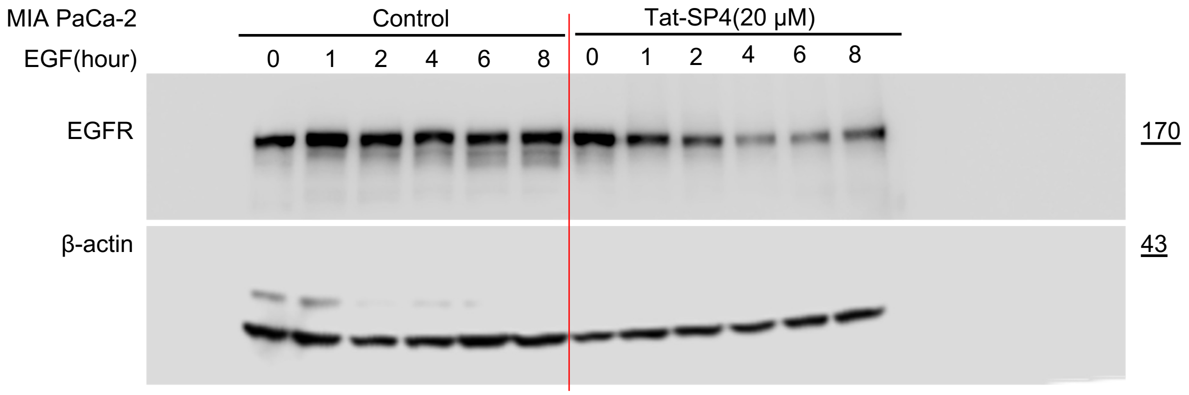

Trial 2

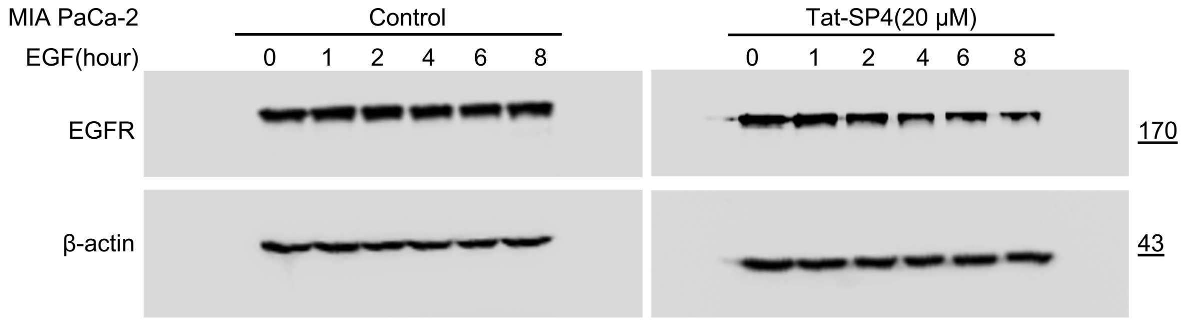

Trial 3

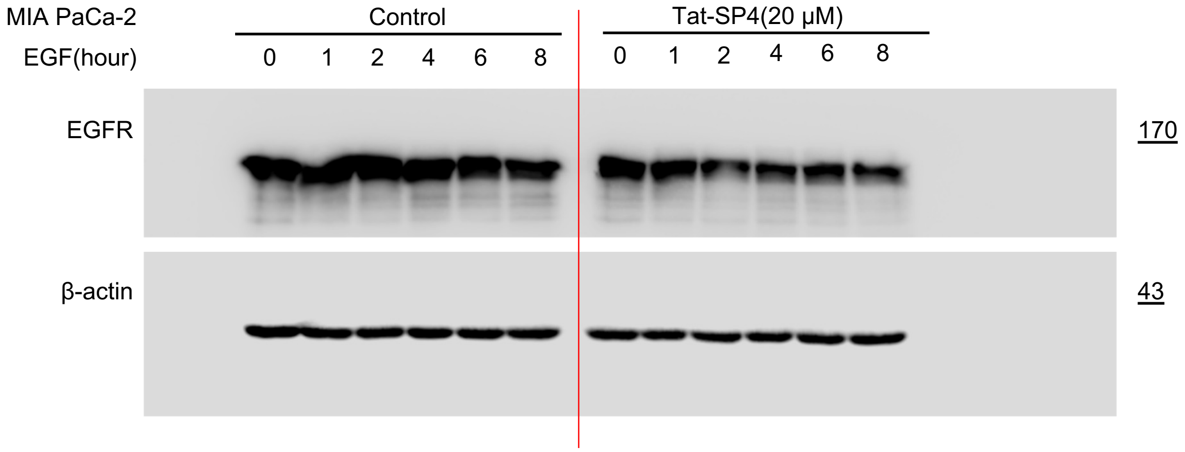

Figure 2E (repeat 3 times)

Trial 1

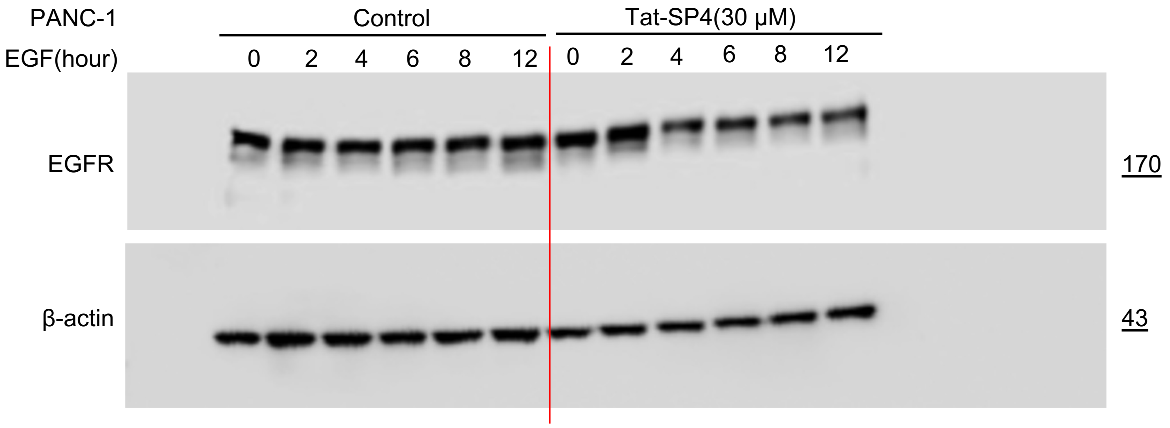

Trial 2

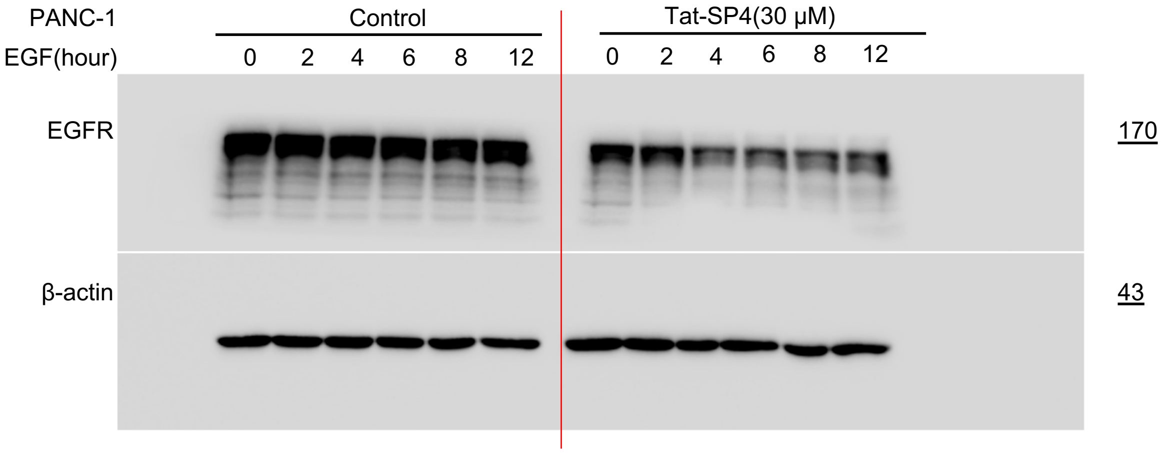

Trial 3

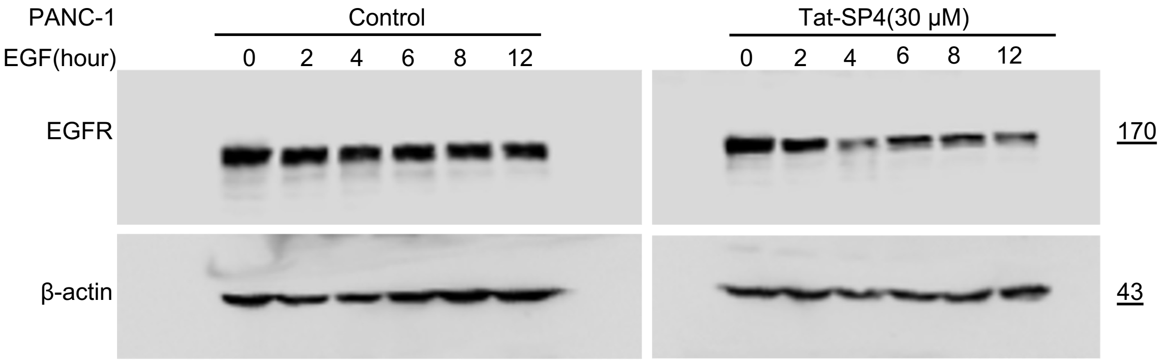

Supplement: Supplementary file 1 [file cancers-15-00953-s001.zip › cancers-2166019-Supplementary.pdf]
